# Supplementary material for: Post-healing follow-up study of patients in remission for diabetic foot ulcers Pied-REM study
Source: PLoS One. 2022 May 19;17(5):e0268242. doi: 10.1371/journal.pone.0268242 (PMC9119502; doi:10.1371/journal.pone.0268242)
Supplement: S1 Checklist — (DOC) [file pone.0268242.s001.doc]

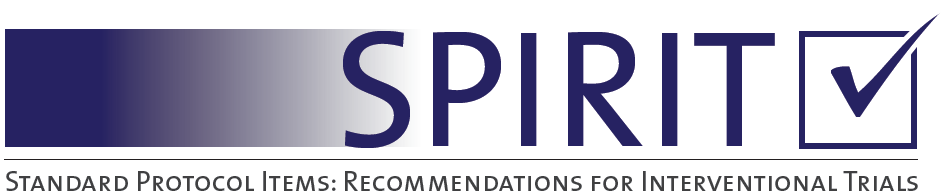


SPIRIT 2013 Checklist: Recommended items to address in a clinical trial protocol and related documents*

| Section/item | ItemNo | Description |  | |
| --- | --- | --- | --- | --- |
| **Administrative information** | | |  | |
| Title | 1 | **POST-HEALING FOLLOW-UP STUDY OF PATIENTS IN REMISSION FOR DIABETIC FOOT ULCERS**  **Pied-REM study** |  | |
| Trial registration | 2a | ClinicalTrials.gov Identifier: NCT04892771 |  | |
|  |  |  |  |  |
| Protocol version | 3 | Version 1.0 of 06/04/2021 |  | |
| Funding | 4 | all financial costs are borne by the sponsor "clinical research unit of the centre hospitalier sud Francilien |  | |
| Roles and responsibilities |  |  |  | |
|  | 5a | **Principal Investigator** Dured DARDARI MD PhD`  Department of Endocrinology, Diabetology and Metabolic Diseases  Centre Hospitalier Sud Francilien  40, Avenue Serge Dassault  91106 Corbeil-Essonnes Cedex, France  Tel: (+33) 1 61 69 40 17  Email address: [dured.dardari@chsf.fr](mailto:dured.dardari@chsf.fr) |  | |
|  | 5b | Collection, management, analysis, and interpretation of data; writing of the report; and the decision to submit the report for publication, are managed by Dr DARDARI  Analysis, and interpretation of data are supported by the sponsor clinical research unit of the centre hospitalier sud Francilien |  | |
|  |  |  |  | |
| Introduction | 1 |  |  | |
| Background and rationale | 1.1 |  |  | |
|  |  |  |  | |
| Objectives | 2 |  |  | |
| Trial design | 2.2 | Non-interventional research study involving  Human people |  | |
| Methods: Participants, interventions, and outcomes | | | 9 | Description of study settings (eg, community clinic, academic hospital) and list of countries where data will be collected. Reference to where list of study sites can be obtained |
| Study setting | 2.3 | - **Inclusion Criteria**  .  patients aged 18 years and over with type 1 or 2 diabetes who receive follow-up care at the Centre Hospitalier Sud-Francilien for healed foot ulcer |  | |
| Eligibility criteria | 2.3 | Pregnant women with gestational diabetes. |  | |
| Interventions | 2.3 | Criteria for discontinuing or modifying allocated interventions for a given trial participant (eg, drug dose change in response to harms, participant request, or improving/worsening disease)/ **Not applicable** |  | |
|  | 2.3 | Strategies to improve adherence to intervention protocols, and any procedures for monitoring adherence (eg, drug tablet return, laboratory tests) **Not applicable** |  | |
|  | 2.3 | Relevant concomitant care and interventions that are permitted or prohibited during the trial **Not applicable** |  | |
|  | 2.3 | Primary, secondary, and other outcomes, including the specific measurement variable (eg, systolic blood pressure), analysis metric (eg, change from baseline, final value, time to event), method of aggregation (eg, median, proportion), and time point for each outcome. Explanation of the clinical relevance of chosen efficacy and harm outcomes is strongly recommended **Not applicable** |  | |
| Outcomes | 2.3 | Time schedule of enrolment, interventions (including any run-ins and washouts), assessments, and visits for participants. A schematic diagram is highly recommended (see Figure) **Not applicable** |  | |
| Participant timeline | 2.3 | 24 months |  | |
| Sample size | 2.3 | 200 participants |  | |
| Recruitment | 2.4 |  |  | |
| **Methods: Assignment of interventions (for controlled trials)** | | |  |  |
| Allocation: |  | Method of generating the allocation sequence (eg, computer-generated random numbers), and list of any factors for stratification. To reduce predictability of a random sequence, details of any planned restriction (eg, blocking) should be provided in a separate document that is unavailable to those who enrol participants or assign interventions/ **Not applicable** |  | |
| Sequence generation |  | Mechanism of implementing the allocation sequence (eg, central telephone; sequentially numbered, opaque, sealed envelopes), describing any steps to conceal the sequence until interventions are assigned **Not applicable** |  | |
| Allocation concealment mechanism |  | Who will generate the allocation sequence, who will enrol participants, and who will assign participants to interventions **Not applicable** |  | |
| Implementation |  | Who will be blinded after assignment to interventions (eg, trial participants, care providers, outcome assessors, data analysts), and how **Not applicable** |  | |
| Blinding (masking) |  | If blinded, circumstances under which unblinding is permissible, and procedure for revealing a participant’s allocated intervention during the trial **Not applicable** |  | |
|  |  | If blinded, circumstances under which unblinding is permissible, and procedure for revealing a participant’s allocated intervention during the trial **Not applicable** |  | |
| **Methods: Data collection, management, and analysis** | | | 18a | Plans for assessment and collection of outcome, baseline, and other trial data, including any related processes to promote data quality (eg, duplicate measurements, training of assessors) and a description of study instruments (eg, questionnaires, laboratory tests) along with their reliability and validity, if known. Reference to where data collection forms can be found, if not in the protocol |
| Data collection methods | 2.4 |  |  | |
|  |  | Plans for data entry, coding, security, and storage, including any related processes to promote data quality (eg, double data entry; range checks for data values). Reference to where details of data management procedures can be found, if not in the protocol **Not applicable** |  | |
| Data management | 20a | Statistical methods for analysing primary and secondary outcomes. Reference to where other details of the statistical analysis plan can be found, if not in the protocol |  | |
| Statistical methods | 2.5 | 2.5 Regarding the sample size, to demonstrate a difference of 25% to 40% between two groups, with a risk of first species of 5%, a power of 80%, and continuity correction, 158 patients are required. We estimate that 200 diabetic patients are currently being monitored at the hospital for multidisciplinary “foot remission” consultations, which should largely be able to enlist the required number of patients. |  |  |
| Consent or assent | 28 | Financial and other competing interests for principal investigators for the overall trial and each study site **Not applicable** |  |  |
|  | 29 | Statement of who will have access to the final trial dataset, and disclosure of contractual agreements that limit such access for investigators **Not applicable** |  |  |
| Confidentiality | | | 30 | Provisions, if any, for ancillary and post-trial care, and for compensation to those who suffer harm from trial participation **Specific documents of non-interventional research involving human subjects will be archived by the investigator and the sponsor for a period of 15 years after the end of the research.** |
| Declaration of interests | 31a | Plans for investigators and sponsor to communicate trial results to participants, healthcare professionals, the public, and other relevant groups (eg, via publication, reporting in results databases, or other data sharing arrangements), including any publication restrictions  **The sponsor must be mentioned as an affiliate of the author(s) of any publications resulting from this research, and the CHSF must be named as sponsor and a copy of work sent to the CRU (see the terms of affiliation and those under which the sponsor must be mentioned below).** |  |  |
| Access to data | 31b | Authorship eligibility guidelines and any intended use of professional writers  **Not applicable** |  |  |
| Ancillary and post-trial care | 31c | Plans, if any, for granting public access to the full protocol, participant-level dataset, and statistical code  **Not applicable** |  |  |
| Dissemination policy |  |  |  |  |
|  | | | 32 | Model consent form and other related documentation given to participants and authorised surrogates  **Not applicable** |
|  | 33 | Plans for collection, laboratory evaluation, and storage of biological specimens for genetic or molecular analysis in the current trial and for future use in ancillary studies, if applicable **Not applicable** |  |  |
| Appendices |  | **Not applicable** |  |  |
| Informed consent materials |  |  |  | |
| Biological specimens |  |  |  | |
|  |  |  |  | |
|  |  |  |  | |
|  |  |  |  | |
|  |  |  |  | |
|  |  |  |  | |
|  |  |  |  | |
|  |  |  |  | |
|  |  |  |  | |
|  |  |  |  | |
|  |  |  |  | |

*It is strongly recommended that this checklist be read in conjunction with the SPIRIT 2013 Explanation & Elaboration for important clarification on the items. Amendments to the protocol should be tracked and dated. The SPIRIT checklist is copyrighted by the SPIRIT Group under the Creative Commons “[Attribution-NonCommercial-NoDerivs 3.0 Unported](http://www.creativecommons.org/licenses/by-nc-nd/3.0/)” license.
