# Supplementary material for: Post-healing follow-up study of patients in remission for diabetic foot ulcers Pied-REM study
Source: PLoS One. 2022 May 19;17(5):e0268242. doi: 10.1371/journal.pone.0268242 (PMC9119502; doi:10.1371/journal.pone.0268242)
Supplement: S1 Fig — (DOC) [file pone.0268242.s002.doc]

**Figure**. POST-HEALING FOLLOW-UP STUDY OF PATIENTS IN REMISSION FOR DIABETIC FOOT ULCERS Pied-REM study

|  | **STUDY PERIOD** | | | | | | | |
| --- | --- | --- | --- | --- | --- | --- | --- | --- |
|  | **Enrolment** | **Allocation** | **Post-allocation** | | | | | **Close-out** |
| **TIMEPOINT**** | ***-t_1_*** | **0** | ***t_1_*** | ***t_2_*** | ***t_3_*** | ***t_4_*** | ***etc.*** | ***t_x_*** |
| **ENROLMENT:** |  |  |  |  |  |  |  |  |
| **Eligibility screen** |  |  | X |  |  |  |  |  |
| **Informed consent** |  |  | X |  |  |  |  |  |
| ***[List other procedures]*** |  |  | X |  |  |  |  |  |
| **Allocation** |  |  |  |  |  |  |  |  |
| **INTERVENTIONS:** |  |  |  |  |  |  |  |  |
| ***[Intervention A]*** |  |  |  |  |  |  |  |  |
| ***[Intervention B]*** |  |  |  |  | X |  |  |  |
| ***[List other study groups]*** |  |  |  |  |  |  |  |  |
| **ASSESSMENTS:** |  |  |  |  |  |  |  |  |
| ***[List baseline variables]*** |  |  |  | X |  | X |  |  |
| ***[List outcome variables]*** |  |  |  | X |  | X |  |  |
| ***[List other data variables]*** |  |  | X | X | X | X |  |  |

*Recommended content can be displayed using various schematic formats. See SPIRIT 2013 Explanation and Elaboration for examples from protocols.

**List specific timepoints in this row.
