# Supplementary material for: Post-healing follow-up study of patients in remission for diabetic foot ulcers Pied-REM study
Source: PLoS One. 2022 May 19;17(5):e0268242. doi: 10.1371/journal.pone.0268242 (PMC9119502; doi:10.1371/journal.pone.0268242)
Supplement: S1 File — (DOCX) [file pone.0268242.s003.docx]

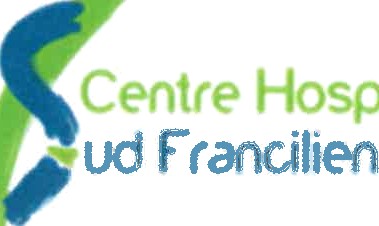


« Study of post-healing follow-up in people in remission of a diabetic foot wound»

Pied-REM

RESEARCH PROTOCOL NOT INVOLVING THE HUMAN PERSON

Version N°1.0 du 19/04/2021 Project code: 2021/0027

###### Investigator Coordinator:

**Scientific Director:**

**Sponsor** :

**Marie BOULY**

Department of Endocrinology,Diabetology and Metabolic Diseases Unit

Centre hospitalier Sud Francilien 40, avenue Serge Dassault 91106 Corbeil-Essonnes cedex

Tél : 06 18 49 14 93

Mail : [marie.bouIy@chsf.fr](mailto:marie.bouIy@chsf.fr)

**Dr Dured DARDARI**

Department of Endocrinology,Diabetology and Metabolic Diseases Unit

Centre hospitalier Sud Francilien 40, avenue Serge Dassault 91106 Corbeil-Essonnes cedex

Tél : 01 61 69 40 17

Mail : [dured.dardari@chsf.fr](mailto:dured.dardari@chsf.fr)

**Centre Hospitalier Sud Francilien** Clinical Research Unit (URC)

40 Avenue Serge Dassault

91106 Corbeil-Essonnes Cedex

Responsable URC : Elodie HENRY

Tél : 01 61 69 37 30 - Mail : elodie.henry@chsf.fr

**Suivi projet** : Caroline TOURTE

Tél : 01 61 69 31 50 — Mail : [caroline.tourte@chsf.fr](mailto:caroline.tourte@chsf.fr)

###### Methodologist-Biostatistician: Dr François-Xavier LABORNE

Centre Hospitalier Sud Francilien Clinical Research Unit (URC)

40 avenue Serge Dassault 91106 Corbeil-Essonnes Cedex Mail : fx.Iaborne@samu91.oro

1/14

SIGNATURE PAGE FOR A RESEARCH PROTOCOL THAT DOES NOT INVOLVE THE HUMAN PERSON

Research Code : 2021/0027

Title : Study of post-healing follow-up in people in remission of a diabetic foot wound

Version 1.0 of 19/04/2021

| **Investigator Coordinator** : | |
| --- | --- |
| Marie BOULY  Department of Endocrinology, Diabetology ans Metabolic diseases  Centre Hospitalier Sud Francilien | Date : ... 30/04/2021  Signature : |
| **Sponsor** | |
| **Centre Hospitalier Sud Francilien**  Mélanie JULLIAN  Unité de Recherche Clinique 40 Avenue Serge Dassault  91106 Corbeil-Essonnes Cedex | Date : 29/04/2021  Signature : |


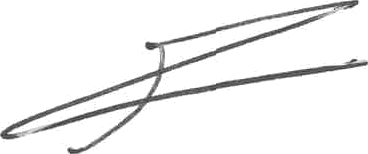
The research will be conducted in accordance with the protocol and the applicable legislative and regulatory provisions.

TABLE OF CONTENTS

i. ABSTRACT 4

1. [OBJECTIVES 9](#_TOC_250012)

1.1. PRIMARY OBJECTIVE…………………………………………………………………………………………………………...9

1.2. SECONDARY OBJECTIVES……………………………………………………………………………………………………...9

1. [METHOD AND POPULATION. 9](#_TOC_250011)
   1. OUTCOME 9
   2. TYPE OF STUDY 9
   3. STUDY PERIOD. 10
   4. [NUMBER OF PARTICIPATING CENTRES 10](#_TOC_250010)
   5. SUBDJECTS [IDENTIFICATION 10](#_TOC_250009)
   6. SOURCES OF PERSONNAL DATAS 10
   7. POPULATION STUDIED. 10
   8. CONDUCT OF THE RESEARCH 10
2. [DATA MANAGEMENT. 11](#_TOC_250008)
   1. METHOD OF DATA COLLECTION 1l
   2. DATA CIRCUIT, PROTECTION AND CONFIDENTIALITY. 1 l
   3. DOCUMENTS AND DATA RETENTION. 1 l
3. [STATISTICAL ASPECTS. 12](#_TOC_250007)
   1. STATISTICAL JUSTIFICATION OF SAMPLE SIZE 12
   2. DESCRIPTION OF STATISTICAL METHODS 12
4. [ETHICAL AND LEGACY ASPECTS. 12](#_TOC_250006)
   1. ROLE OF THE SPONSOR. 12
   2. METHODS OF INFORMING THE RELEVANT POPULATION. 12
   3. PROCEDURES FOR COMPUTER REGULATION AND FREEDOM 13
   4. REQUEST FOR OPINION OF THE ETHICS COMMITTEE 13
   5. RESPONSIBILITY OF THE INVESTIGATOR TOWARDS THE SPONSOR. 13
   6. [FINAL REPORT 13](#_TOC_250005)
5. [RULES CONCERNING PUBLICATION 13](#_TOC_250004)
   1. [MENTION OF CHSF AFFILIATION FOR PROJECTS PROMOTED BY CHSF 13](#_TOC_250003)
   2. MENTION OF CHSF SPONSOR IN THE MANUSCRIPT ACKNOWLEDGMENTS. 13
6. **RESUME**

| **Full title** | Study of post-healing follow-up in people in remission of a diabetic foot wound | | |
| --- | --- | --- | --- |
| **Acronym** | Pied-REM | | |
| **Investigator coordonnator** | Marie BOULY  Endocrinology,Diabetology and Metabolic Diseases Unit  Centre hospitalier Sud Francilien 40, avenue Serge Dassault 91106 Corbeil-Essonnes cedex  Tél : 06 18 49 14 93  Mail : [marie.bouly@chsf.fr](mailto:marie.bouly@chsf.fr) |  |  |
| **Scientific Director** | Dr Dured DARDARI  Endocrinology,Diabetology and Metabolic Diseases Unit  Centre hospitalier Sud Francilien 40, avenue Serge Dassault 91106 Corbeil-Essonnes cedex  Tél : 01 61 69 40 17  Mail : dured.dardari@chsf.fr |  |  |
| **Sponsor** | Centre Hospitalier Sud Francilien | | |
| **Scientific justification** | The rate of recurrence of diabetic foot wounds is 60% at 3 years. We would like to examine the link between post-healing follow-up and recurrence. | | |
| **Objectiv and primary outcome** | Primary objectiv : Evaluate the benefit of a specific multidisciplinary follow-up of the healed diabetic foot at the rate of 2 annual consultations on wound recurrence.  Primary outcome: Foot wound recurrence at 2 years | | |
| **Objectives and secondary outcomes** | Secondary objectives :  Evaluate the benefit of a specific multidisciplinary follow-up of the healed diabetic foot at the rate of 2 annual consultations on :   - recurrence with the need for hospitalization. - pedicure follow-uo of patients. - wearing suitable footwear.   Secondary outcomes   - Hospitalization for foot wound at 2 years - Pedicure follow-up at 2 years - Wearing suitable footwear at 2 years | | |
| **Type of study** | Monocentric retrospective cohort study  observational study. | | |

| **Inclusion criteria** | - Patient with a type 1 or 2 follow-up at the Centre Hospitalier Sud-FranciIien (CHSF), - Discharge from hospital from a foot wound between 2017 and 2019 with a 2 years post-healing follow-up - Age greater than or equal to 18 years, - People not deprived of liberty, - Subject informed of the study and did not object. |
| --- | --- |
| **Non-inclusion criteria** | Patient refusing the study. |
| **Information for participants** | Individual patient information |
| **Number of subjects**  **Selected** | 158 patients |
| Number of centers | see addenda n°1 |
| Duration of the research | Period of time for which data is available : 2017-2021 Duration of the research: 1 year |

#### 1.Expected outcomes and opportunities / Hypothèses/ Justification of public interest

We hypothesize that 2 annual multidisciplinary consultations in diabetic patients with a healed foot ulcer ensure optimal follow-up and minimize the risk of recurrence and hospitalization. In our experience, such a follow-up limits the rate of recurrence at 1 year to about 25%, compared to 40% of recurrence of diabetic foot ulcers with a conventional follow-up. Such a result would have a definite benefit in terms of health care expenses (treatments, hospitalization, work stoppages).

#### OBJECTIVES

- 1. **Primary objectiv**

To evaluate the benefit of a specific multidisciplinary follow-up of the healed diabetic foot at the rate of 2 annual consultations on wound recurrence.

#### Secondary objectives

Evaluate the benefit of a specific multidisciplinary follow-up of the healed diabetic foot at the rate of 2 annual consultations on :

- recurrence with the need for hospitalization.
- pedicure follow-uo of patients.
- wearing suitable footwear.

#### METHOD ET POPULATION

- 1. **Outcomes**

#### Primary outcome

Foot wound recurrence at 2 years

#### Secondary outcomes

- Hospitalization for foot wound at 2 years
- Pedicure follow-up at 2 years
- Wearing suitable footwear at 2 years
  1. Type of study

Monocentric retrospective cohort study, observational study.

9/î4

###### Duration of the research

Period of time for which data is available : 2017-2021

Duration of the research: 1 year

###### Number of centers

Monocentric research — cf. addenda n°1

###### Subjects identification

In this research, subjects will be identified as follows :

n° centre (3 numéric positions) — order of selection of the person in the center (4 numéric positions ) - initial name - initial first name '

I_0_I_1_I - I_0_I_0_I_0_I_1_I - I_N_I_P_I

This reference is unique and will be kept for the duration of the research.

###### Sources of personnal data

Data will be collected from patients' medical records.

###### Study population

- - 1. **Inclusion criteria**
- Patient with a type 1 or 2 follow-up at the Centre Hospitalier Sud-FranciIien (CHSF),
- Discharge from hospital from a foot wound between 2017 and 2019 with a 2 years post-healing follow-up
- Age greater than or equal to 18 years,
- People not deprived of liberty,
- Subject informed of the study and did not object.

###### Non-inclusion criteria

- Patient refusing the study.

###### Conduct of the research

Eligible patients are selected from the computerized database of the diabetes department of the CHSF, over the period 2017-2019. Eligibility criteria are then verified in the patient record.

After sending the information form to the patients and respecting the no-objection period, the patients will be included in the study and the data collection will be performed.

The data are collected from the patient records, and are recorded on a computer medium.

Each patient will be assigned a group, either the experimental group or the control group. The 2 groups will be defined according to the number of consultations honored each year over a 2-year period Patients who have honored at least an average of 2 consultations per year for 2 years, i.e. 4 sessions over 2 years at least, will be included in the experimental groupPatients who do not reach this number will be included in the control group.

t0/14

###### DATA MANAGEMENT

- 1. **Data collection methods**

The data will be collected retrospectively, via the computerized medical records, on the Reference software.

###### Data flow and confidentiality protection method

- - 1. **Flow**

Data will be collected by the investigating team in a pseudo-anonymized manner in a password protected Excel file hosted on the hospital network

###### Nature of data collected

- Verification of inclusion criteria

- Age and gender

- Type and duration of diabetes

- Presence of arterial disease, diabetic neuropathy

- History of amputation

- HbAlC

During the 2-year observation period from the date of recovery:

- Number of follow-up visits honored over a two-year period from the time of healing

- Recurrence of foot wounds Hospitalization for foot wounds

- Pedicure follow-up

- Appropriate footwear.

###### Confidentiality

During or at the conclusion of the research, the data collected on the individuals who are the subject of the research and transmitted to the sponsor by the investigators (or any other specialized parties) will be rendered non-identifying.

Under no circumstances may the names of the persons concerned or their addresses appear in clear text..

Only the initials of the surname and first name will be recorded, accompanied by a coded number specific to the research indicating the order of inclusion of the subjects.

The sponsor will ensure that each person who takes part in the research has given his or her consent to access individual data concerning him or her and strictly necessary for the quality control of the research.

###### Retention of documents and data

Research documents and data will be retained for up to 2 years after publication.

11/14

###### STATISTICAL ASPECTS

- 1. **Statistical justification of the sample size**

This retrospective study does not require a specific size calculation.

###### Description of the statistical methods

The 2 groups will be defined according to the number of consultations honored each year over a 2-year period. Patients who have attended at least an average of 2 consultations per year for 2 years, i.e. a minimum of 4 sessions over 2 years, will be included in the experimental group Patients who do not reach this number will be included in the control group.

The categorical variables will be presented by their numbers and percentages, and compared by Fisher's exact tests or chi2. Quantitative variables will be presented by their median and interquartile range, or by their mean and standard deviation, and compared by t-tests or Wilcoxon tests, depending on their distribution

The rates of recurrence, hospitalization, pedicure follow-up and adapted footwear will be compared between the 2 groups by Fisher exact tests.

The time to recurrence in each group will be measured by the Kaplan-Meier estimator, and compared between the 2 groups by a log-rank test. If the conditions for application are met, the time to recurrence will be analyzed by a multivariate Cox regression model taking into account the characteristics of the patients (age, sex, length of diabetes, presence of arterial disease, neuropathy) as well as whether or not they were followed up in a "foot in remission" consultation, whether they had pedicures and whether they wore adapted shoes.

The results of the multivariate Cox regression study took into account the characteristics of the patients (age, sex, length of diabetes, presence of arterial disease, neuropathy) as well as whether or not they were followed up in a "foot in remission" consultation, whether or not they had received pedicure and whether or not they wore adapted shoes.

All tests will be performed in a two-sided manner, with a 1st species risk alpha fixed at 5%. The statistical analysis will be performed by Dr François-Xavier Laborne of the Clinical Research Unit of the CHSF, using the R software (R Core Team (2021). R: A language and environment for statistical computing. R Foundation for Statistical Computing, Vienna, Austria. [https://www.R-project.org/](http://www.R-project.org/))).

###### ETHICAL AND LEGAL ASPECTS

- 1. **Rôle of the sponsor**

The Centre Hospitalier Sud Francilien is the sponsor of this research.

The promoter CHSF reserves the right to definitively suspend the inclusions, at any time, if it appears that the inclusion objectives are not met.

###### Methods of informing the population concerned

Each patient will be informed by an information note that he/she will receive at home. In case of no response from the patient or no refusal within 15 days after the letter is sent, the patient will be considered to be in favor of inclusion in the study..

12/14

Protocole Pied-REM ». version 1.0 du 19/04/2021

#### Procedures relating to data processing and freedom regulations

##### The computer file used for this research is implemented in accordance with French (amended Data Protection Act) and European (General Data Protection Regulation - GDPR) regulations.

This research does not fall within the framework of the "Reference Methodology" (MR-004) of the CNIL because it is a monocentric study.

The data processing implemented in the framework of this research will be registered in the CHSF register, under the responsibility of the CHSF Data Protection Officer, in accordance with the provisions of the RGPD (General Data Protection Regulation).

- 1. **Requesting an opinion from the Ethics Committee**

An ethical opinion was sought from the CHSF Ethics Committee.

#### Investigator’s responsibilities to the sponsor

##### The coordinating investigator or qualified individual agrees to provide the sponsor with information regarding the status of the research.

Any changes to the protocol and/or the research information note should be submitted to the sponsor.

##### **Final research report**

The report will be completed within 12 months of the last data collection.

1. **RULES FOR PUBLICATION**
   1. **Mention of the affiliation of the CHSF for projects promoted by the CHSF**

- If an author has several affiliations, the order in which the institutions are cited (CHSF, University, INSERM...) does not matter
- Each of these affiliations must be identified by an address separated by a semicolon
- The CHSF establishment must appear under the acronym "**Centre Hospitalier Sud Francilien"** first in the address followed precisely by : **Center Hospitalier Sud Francilien**, department, city, postal code, France
  1. **Mention of the CHSF sponsor in the ”acknowledgments” of the manuscript**

##### ”The sponsor was Centre Hospitalier Sud Francilien »

This research will be recorded on the website <http://cIinicaItriaIs.qov/>

13/14

Protocole Pied-REM », version 1.0 du 19/04/2021
