# Supplementary material for: Post-healing follow-up study of patients in remission for diabetic foot ulcers Pied-REM study
Source: PLoS One. 2022 May 19;17(5):e0268242. doi: 10.1371/journal.pone.0268242 (PMC9119502; doi:10.1371/journal.pone.0268242)
Supplement: S2 File — (PDF) [file pone.0268242.s004.pdf]

Corbeil le 18 Mai 2021

Consultation Éthique des protocoles de recherche STOPPOMPE, TLS DIAB, THROM-PP, PIED-REM et 2021 en promotion du CHSF

Membres du comité d'éthique du CHSF : Dr Célia Salanoubat, Mme karine Dufau, Mr Steven Lagadec

PIED-REM v0.1

Investigateur promoteur : Marie Bouly

Responsable scientifique : Dr Dured Dardari

Design : Étude de cohorte rétrospective monocentrique observationnelle

Taille de l'échantillon : 158 patients

Avis du comité d'éthique : **FAVORABLE**

Étude rétrospective sur des données collectées en dehors du projet de recherche. Le caractère inoffensif de l'étude est implicitement établi et le comité d'éthique n'aura donc pas à se prononcer sur la sécurité des personnes qui se prête aux expérimentations. Cependant le comité émet quelques remarques mineures sur le protocole et ses annexes :

- Conformément aux articles 13 et 14 du RGPD, les coordonnées du délégué à la protection des données du responsable de traitement doivent être indiquées dans la note d'information des personnes.

- Le protocole détaille que les dates d'inclusion vont de 2017 à 2019 avec un suivi de 2 ans et donc jusqu'en 2021. Ce détail peut laisser penser qu'une partie du suivi se fera déroule de façon prospective.

- La note d'information est par endroit rédigée en des termes trop techniques. Exemple : « Le recueil des données sera rétrospectif »

- Dans le protocole de l'étude il n'est pas précisé que la filiation à un régime de sécurité social était un « Critère d'inclusion » ou par opposition un « Critère de non inclusion »

- Le consentement par défaut, bien qu'acceptable pour ce design d'étude est toujours éthiquement inapproprié d'autant que la limite de 15 jours semble être un délai court pour une population composée de sujets malades possiblement hospitalisée au moment de l'envoi du courrier. Un consentement verbal par contact téléphonique pourrait compléter la démarche et nous assurer que l'information a bien été comprise.
