# Supplementary material for: Post-healing follow-up study of patients in remission for diabetic foot ulcers Pied-REM study
Source: PLoS One. 2022 May 19;17(5):e0268242. doi: 10.1371/journal.pone.0268242 (PMC9119502; doi:10.1371/journal.pone.0268242)
Supplement: Attachment — Submitted filename: Response to Reviewers plose one .docx [file pone.0268242.s005.docx]

Response to Reviewers

Dear editor and reviewers:

Many thanks for your comments and for the interest you have given to our manuscript please find the answers to your remarks

**Reviewer #1**

1 - Correct the world “Mai” to “May” in the Introduction: Done ( we are very sorry for this mistake)

2- "The study protocol was approved by" was mentioned twice in same page, in the Introduction and in the Description of study protocol. Possibly to remove it from the Introduction : modification done

3- In the Study description, it was mentioned that the study is a retrospective observational and the part mentioned in Data collection about collecting patients files and reviewing their cases seems appropriate with the definition of this study. But also mentioned in the Data collection that there will be a 2-year observation period from the date of inclusion with different variables intended to be measured which seems to be a different period than the initial one described. So, will there be a prospective observational part in this study? If yes, then it needs to be mentioned clearly in the Description of study protocol.

thank you very much for your comment, the study is completely retrospective with an observation period of 2 years post healing of a foot in a living diabetic patient, there is no prospective component

4- Mentioned in the Statistical methods that there are two groups, experimental and control based on the number of visits, this study protocol needs to be mentioned in the Description of study protocol, not in the Statistical methods.

Modification done

5- - It seems from the Statistical method that the measured outcome is based on having or not having an intervention which is the number of visits or sessions over 2 years between the two groups (experimental and control). That has not been clearly defined in the Study objectives.

Modification done

6-There are study protocol description information in the discussion that needs to placed in the material and methods and better to review and place needed information in appropriate place. For example: “The 2-year follow-up period will allow for a sufficient number of patients to be included: the first patients will be monitored from 2017 to 2019 and the last ones from 2019 to 2021”, this sentence belongs to Material and methods

Modification done

**Reviewer # 2**

1-In the part of the abstract, the author mentioned that ,The rate of recurrence for diabetic foot ulcer (DFU) is 50% at 2 years. For this statement need to add the reference.( what is the source of information).

Reference added

2-In the part of Introduction, study objective part the author mention the word the multidisciplinary
follow-up of healed DFU patients, but did not explore what author mean by multidisciplinary

Thank you very much for your comment, the explanation has been added

3-In the part of Participants- the author did not mention the total number of sample size, Gender of the Participants, and average age of the participants.

thank you very much for your comment, at the current state it is proposed that the study design the results of patient demographics will be proposed in the final publication, thank you for your understanding

4-The study only based on Quantitative methodology. If the authors conduct the study by mixed methodology (quantitative and qualitative) its would more efficient to explore the socio-economic reasons/ factors behind the yearly follow-up for the experimental group and explore the socio-economic reasons/ factors behind are barriers are works for the yearly follow-up for the control group.

thank you very much for this comment, we are going to make an amendment to study the socio-economic reasons associated with the recurrence of wounds
